# Meet the editorial board members: Stephanie Lo and Robert Paulino-Ramírez

**DOI:** 10.1186/s44263-023-00011-8

**Published:** 2023-07-31

**Authors:** Stephanie Lo, Robert Paulino-Ramírez

**Affiliations:** 1 Parasites and Microbes, Wellcome Sanger Institute, Wellcome Genome Campus, Hinxton, UK; 2 Instituto de Medicina Tropical & Salud Global, Universidad Iberoamericana (UNIBE), Santo Domingo, Dominican Republic

## Abstract

In this Q&A, Stephanie Lo and Robert Paulino-Ramírez answer questions about their research fields and share insights into their role as editorial board members at the journal.

## What is the focus of your research and what drew you to this field?

*Stephanie Lo*: Being trained as a clinical microbiologist, I was drawn to the pathogen genomics field as I think genomics holds a great promise to improve global health through reducing infectious diseases. My research focuses on analyzing large-scale genomic data to understand how pathogens evolve to evade vaccines and antibiotics so as to inform more effective infectious disease prevention and treatment policy. Currently, I am leading the Global Pneumococcal Sequencing (GPS) project (https://www.pneumogen.net/gps/). By analyzing tens of thousands pneumococcal genomes worldwide, we inform the design of the next generation of vaccines to further reduce child death caused by a major bacterial killer, *Streptococcus pneumoniae*.

*Robert Paulino-Ramírez*: For many years, I have conducted research in the area of medical virology; my professional training brought me closer to virology from the laboratory bench to the clinical bedside. During my training, I had the opportunity to learn a lot about the care system for people living with HIV and reflect on the many barriers that affect the fact that even today we cannot end a 40-year pandemic. It is since then that I decided to try to answer from science the great challenges that HIV poses for global health.

## What are the main problems that need addressing in the field?

*SL*: Infections caused by common bacteria such as pneumococci are still one of the major killers, especially among young children in low- and middle-income countries (LMICs). To reduce child death, we need to fill in the data gap in LMICs and generate evidence to inform vaccine design and treatment. Whole-genome sequencing is a powerful way to generate interoperable data at scale. In the last three decades, we have accumulated vast experience and expertise to extract public-health relevant information from genome data for pathogen surveillance. Generating genome data is no longer a bottleneck, training more scientists, especially those from LMICs, to analyze and interpret the data is key to generate actionable information to combat infectious diseases in countries where disease burden is highest.

*RPR*: Despite advances in prevention strategies such as pre-exposure prophylaxis (PrEP) and postexposure prophylaxis (PEP), new HIV infections continue to occur. Ensuring that prevention strategies are widely available, affordable, and accessible to all populations at risk, including key populations such as men who have sex with men, people who inject drugs, sex workers, and transgender individuals, is critical to reducing new infections. HIV-related stigma and discrimination continue to be major challenges. People living with HIV often face discrimination, social ostracism, and loss of employment, housing, and social support. Stigma can also prevent individuals from seeking HIV testing, treatment, and care and can contribute to mental health issues and reduced quality of life.

## How do you expect this field to develop in the next few years?

*SL*: In the next few years, genomics will be integrated into pathogen surveillance in many more countries. The research community will be more interconnected to detect outbreak, emerging pathogens, and transmissions. Governance and regulations for data sharing are needed to enhance collaboration and avoid conflicts. It is important to build a practical and transparent system for the community to comply with data sharing policies.

*RPR*: While progress has been made in many parts of the world, there are still regions with high HIV burdens where access to HIV prevention, testing, treatment, and care is limited. This reality is mostly affecting people on the global south. Strengthening global efforts to address the HIV epidemic, including increasing resources for HIV programs, building healthcare capacity, and addressing social and structural barriers, is critical for achieving the goal of ending AIDS as a public health threat by 2030, as outlined in the UNAIDS 95–95-95 targets. While antiretroviral therapy (ART) has transformed HIV from a life-threatening condition to a manageable chronic illness, a cure for HIV remains elusive. Achieving a functional cure or long-term remission where individuals can control the virus without the need for lifelong ART is an area of active research and remains a significant challenge.

## What are you most excited about in your role as an editorial board member for the journal?

*SL*: I am excited about being an editorial board member of BMC Global and Public Health, because this role provides me with a unique opportunity to observe and contribute to progress made to improve global and public health. The compilation of knowledge requires new generations of scientists; I am eager to see new perspectives injected by young scientists to improve human health.

*RPR*: As a global south scientist based in the Caribbean, I believe that I can add a decolonizing vision to the handling and discussion of manuscripts submitted to the journal. This proposes a more inclusive perspective of its contents and brings to the global scientific discussion a vision that reflects what our societies are today. Additionally, this feature enables me to distribute information among a panel of esteemed professionals who are renowned experts in their respective fields and serve as members of the editorial committee. This grants me the opportunity to derive satisfaction from the sometimes-tedious endeavor of comprehending the latest scientific literature.

## Data Availability

Not applicable.

